# In Vitro Antioxidant and In Silico Evaluation of the Anti-β-Lactamase Potential of the Extracts of *Cylindrospermum alatosporum* NR125682 and *Loriellopsis cavenicola* NR117881

**DOI:** 10.3390/antiox13050608

**Published:** 2024-05-16

**Authors:** Albert O. Ikhane, Siphesihle Z. Sithole, Nkosinathi D. Cele, Foluso O. Osunsanmi, Rebamang A. Mosa, Andrew R. Opoku

**Affiliations:** 1 Department of Biochemistry and Microbiology, University of Zululand, KwaDlangezwa 3886, South Africa; sitholezama9476@gmail.com (S.Z.S.); melucy.cele@gmail.com (N.D.C.); opokua@unizulu.ac.za (A.R.O.); 2 Department of Biochemistry, Genetics and Microbiology, University of Pretoria, Hatfield 0028, South Africa; rebamang.mosa@up.ac.za

**Keywords:** β-lactamase, cyanobacteria, molecular docking, antioxidants, GC-MS

## Abstract

Cyanobacteria in recent times have been touted to be a suitable source for the discovery of novel compounds, including antioxidants and antibiotics, due to their large arsenal of metabolites. This study presents the in vitro antioxidant and in silico evaluation of *Cylindrospermum alatosporum* NR125682 and *Loriellopsis cavenicola* NR117881, isolated from freshwater ponds around the campus of the University of Zululand, South Africa. The isolates were confirmed using 16S rRNA. Various crude extracts of the isolated microbes were prepared through sequential extraction using hexane, dichloromethane, and 70% ethanol. The chemical constituents of the crude extracts were elucidated by FTIR and GC-MS spectroscopy. The antioxidant potential of the extracts was determined by the free radical (DPPH, ABTS, ^•^OH, and Fe^2+^) systems. Molecular docking of the major constituents of the extracts against β-lactamase was also evaluated. GC-MS analysis indicated the dominating presence of n-alkanes. The extracts exhibited varying degrees of antioxidant activity (scavenging of free radicals; an IC_50_ range of 8–10 µg/mL was obtained for ABTS). A good binding affinity (−6.6, −6.3 Kcal/mol) of some the organic chemicals (diglycerol tetranitrate, and 2,2-dimethyl-5-(3-methyl-2-oxiranyl)cyclohexanone) was obtained following molecular docking. The evaluated antioxidant activities, coupled with the obtained docking score, potentiates the antimicrobial activity of the extracts.

## 1. Introduction

The troubling rise of antibiotic resistance microbes has led to cascades in drug development research. Research is shifting towards molecules that effect destruction through new pathways or novel cellular targets in the battle against resistant bacteria [1]. Furthermore, combinational therapy of antioxidants and antibiotics is currently being explored to destroy resistant bacteria and reduce host oxidative stress, and natural products are abundant sources of such compounds [2]. Aiyer, et al. [3] reported the biofilm disruption ability of an antibiotic–antioxidant therapy against *Burkholderia cenocepacia* in cystic fibrosis treatment, suggesting an added antibacterial potential of the tested antioxidants. It is evident that antioxidants are an important aspect of medicinal health [4].

Antioxidants are utilised in biological systems to reduce the overaccumulation of oxidative species such as reactive oxygen species (ROS) and reactive nitrogen species (RNS) and mitigate oxidative stress [5]. ROS are known to be exploited by macrophages for the destruction of pathogens during infection [6]; ROS are usually induced in a pro-oxidative manner—induce oxidative stress through the generation of oxidants—resulting in lipid peroxidation that disrupts the cellular membrane of pathogens, eventually leading to apoptosis. ROS action has also been described as a method by which some antibiotics (such as aminoglycosides and quinolones) effect cellular damage [7]; such antibiotics promote the accumulation of OH^•^ and H_2_O_2_ in electron transfer to O_2_ during aerobic respiration [8]. However, bacteria antioxidant enzymes such as superoxide dismutase (SOD) and catalases are highly effective at scavenging residual H_2_O_2_ [9]. Bacteria also possess complex gene regulator systems adapted to produce other antioxidant proteins [10]. Such antioxidant defence system coupled with antibiotic resistance mechanisms allows for the persistence of resistant infections in hosts, leading to the domino effect of oxidative stress and hyperinflammation [11]. Nature have always developed unique and intriguing molecules that science has exploited in medicine; it is no surprise that the search for novel drugs greatly involves the screening of natural organisms. Their low toxicity and high stability give them an advantage over synthetic drugs [12]. Recently, research has shifted towards microbes as promising sources of novel natural products.

Cyanobacteria are among a diverse group of photosynthetic prokaryotes that have been around for a significantly long period and evolved to colonise a variety of habitats, possess a myriad of metabolites that allow for their efficient survival, and possess photosynthetic pigments that allows for autotrophic energy creation [13]. Photosynthetic pigments embedded on thylakoidal membranes allow for energy creation through both photosystems, which leads to a high degree of cellular adaptability and protection to photo-oxidative damage due to their daily exposure to ultraviolet radiation (UVR) [14]. Cyanobacteria also possess a series of defences against oxidative damage [15]. The recent literature has brought to light the myriad of metabolites that cyanobacteria produce [16], which can thus be explored for the development of antioxidants and antibiotics.

Beta-lactamases are hydrolytic bacterial enzymes with a profound affinity for hydrolysing the lactam ring of β-lactam drugs, the inhibition of these enzymes form a crucial area in the reduction of antibiotic resistance, as β-lactam are a very important antibiotic class [17]; β-lactamases employ two strategies to hydrolytically attack the β-lactam functional group of penicillin and cephalosporins, thereby inactivating the antibiotic. One way is through the action of an active ring-opening serine (Ser) nucleophilic attack. The other hydrolytic mechanism is achieved through the activation of water through a Zn^2+^ centre, which facilitates the nucleophilic attack of the β-lactam carbonyl carbon [18]. Based on these mechanisms, β-lactamases have been categorised into two main groups: the serine- β-lactamases and the metallo-β-lactamases [19]; β-lactamases employ water as a co-enzyme in drug destruction and can be excreted to intercept the antibiotic. β-lactamases have thus become a critical target for novel antibiotic development; compounds that can inhibit these enzymes are often deployed in combinations with β-lactams to improve the lethality of the drug.

South Africa is home to a diverse range of habitats; in this study, we report the isolation and identification of *Cylindrospermum alatosporum* NR125682 and *Loriellopsis cavenicola* NR117881 from a freshwater pond. Crude extracts from the two cyanobacteria were screened for their chemical properties, and their antioxidant potentials were evaluated. Furthermore, the potential antimicrobial activity of the crude extracts was evaluated using computational analysis through the molecular docking of the observed organic chemicals following gas chromatography–mass spectroscopy (GC-MS) analysis against β-lactamase.

## 2. Materials and Methods

### 2.1. Chemical Reagents

All chemicals used were of analytical grade purchased from Sigma-Aldrich Co. LTD (Steinheim, Germany). BioTek SYNERGY HT plate reader (BioTek Instrument, Winooski, VT, USA) was used for all absorbance reading.

### 2.2. Water Samples Collection

To isolate the cyanobacteria, freshwater samples were aseptically collected with sterile plastic containers from freshwater ponds located in the Vulindlela area, KwaZulu-Natal, South Africa. (GPS-28.852140, 31.840121). The collected samples were kept in the dark and on ice to preserve the obtained water samples and reduce microbial activity during transport to the University of Zululand laboratory. The samples were processed within 24 h of sample collection. The ethical clearance (UZREC 171110-030 PGM 2022/16) for the study was obtained from the University of Zululand Ethical committee.

### 2.3. Isolation and Purification

BG-11 enrichment medium was prepared as described by Stanier et al. [20]. The medium consisted of BG-11 (17.6 mM NaNO_3_, 0.22 mM K_2_HPO_4_, 0.3 mM MgSO_4_·7H_2_O, 0.24 mM CaCl_2_·2H_2_O, 0.012 mM citric acid, 0.02 mM ferric ammonium citrate, 0.002 mM Na_2_EDTA·2H_2_O, and 0.18 mM Na_2_CO_3_), erythromycin (10 µg/mL, added to protect the broth against invading bacteria), and trace metal mix (TMM) (composed of 46 mM boric acid, 9 mM manganese chloride tetrahydrate, 0.77 mM zinc sulphate heptahydrate, 1.6 mM sodium molybdate dihydrate, 0.3 mM copper sulphate pentahydrate, and 0.17 mM cobalt (II) nitrate hexahydrate) to create a suitable broth growth media. An amount of 220 mL of the enrichment media was inoculated with 15 mL of the previously collected water sample to provide a final volume of 235 mL. The broth was incubated in an orbital shaker under continuous illumination (54.36 μmol photons m^−2^s^−1^), supplied by a cold white fluorescent lamp, with shaking at 180 rpm at 25 °C for 14–21 days (until visible cells were observed). Serial dilutions of 1 mL of stock solution (growth medium containing visible cyanobacteria cells) with 9 mL of sterile saline solutions (0.9%) ensued to provide a logarithmic depression of cyanobacteria cells’ concentration. The serially diluted solutions were poured onto Petri dishes containing the enrichment medium, solidified with 1.5% bacteriological agar, and spread using the spread plate technique [21]. Plates were then incubated for 4 weeks (until enough observable growth was obtained). A series of re-plating was carried out to isolate single and pure colonies.

### 2.4. Identification and Characterisation of Cyanobacteria (16S rRna)

16S rRNA identification was performed for the characterisation of the isolated cyanobacteria with some modifications. Genomic DNA was extracted from the cultures received using the Quick-DNA™ Fungal/Bacterial Miniprep Kit (Zymo Research, Irvine, CA, USA, Catalogue No. D6005). The 16S target region was amplified using OneTaq^®^ Quick-Load^®^ 2X Master Mix (New England Biolabs (Ipswich, MA, USA), Catalogue No. M0486) with the cyano-primers CYA359F (5′GGGGAATCTTCCGCAATGGG-3′), CYA781R (a&b), CYA781Ra (5′-GACTACT GGGGTATCTAATCCCATT-3′), and CYA781Rb (5′-GACTACAGGGGTATCTAATCCCTTT-3′). The PCR products were run on a gel and gel-extracted with a Zymoclean™ Gel DNA Recovery Kit (Zymo Research, Catalogue No. D4001). The extracted fragments were sequenced in the forward and reverse direction (Nimagen, (Nijmegen, The Netherlands) BrilliantDye™ Terminator Cycle Sequencing Kit V3.1, BRD 3-100/1000) and purified (Zymo Research, ZR-96 DNA Sequencing Clean-up Kit™, Catalogue No. D4050). The purified fragments were analysed on an ABI 3500 XL Genetic Analyzer (Applied Biosystems, ThermoFisher Scientific, Waltham, MA, USA). A CLC Bio Main Workbench v 7.6 (Qiagen, Hilden, Germany) was used to analyse the .ab1 files generated by the ABI 3500 XL/ABI 3730 XL Genetic Analyzer, and results were obtained by a BLAST search (NCBI) [22].

### 2.5. Batch Cultivation and Harvest

Two of the identified pure colonies were separately inoculated into separate BG-11 enriched media, prepared in 500 mL conical flasks, for biomass production. They were allowed to incubate for two weeks and harvested (at the growth log phase) through centrifugation at 10,000× *g* for 10 min. The wet cell mass was freeze-dried (SP industries, 6KBTES, Warminster, PA, USA) and stored in brown vials at 8 °C until required for use.

### 2.6. Biomass Extraction

The extraction involved sequential incubation (120 rpm, room temperature) of each freeze-dried sample with each solvent for 24 h ((1:5 *w*/*v*) hexane, dichloromethane, and 70% ethanol). At the beginning, the cell mass was first extracted with hexane for 24 h and then filtered. The obtained residue was further extracted with dichloromethane for another 24 h and filtered. Lastly, the residue was extracted with 70% ethanol (24 h) and filtered. The organic filtrates were concentrated using a rotary evaporator at 30 °C (Heidolph Laborota 4000, Schwabach, Germany), whereas the ethanol extract was freeze-dried. This was performed for each of the freeze-dried cyanobacteria. The concentrated extracts were weighed, re-suspended, and kept in brown vials for further analysis [21].

### 2.7. FTIR Analysis

Fourier transform infrared spectroscopy (FTIR) (Spectrum Two, PerkinElmer, MA, USA) was used to identify functional groups present in the crude extract at room temperature (25–28 °C) at the 370–4000 cm^−1^ spectral range. The functional groups were determined by comparing the peak frequencies with the IR spectroscopy correlation table [23].

### 2.8. GC-MS

Chemical characterisation of cyanobacteria extracts was performed by gas chromatography–mass spectrometry (GC-MS) [24]. An Agilent 7890A (Santa Clara, USA) gas chromatography system coupled with a VL-MSD model 5975C with a triple-axis detector was used. The GC-column profile of the GC-MSD used was Agilent 190915-433: 325 °C: 30 m length × 250 µm diameter × 0.25 µm film thickness. A suitable stationary-phase, eluting solvent (ethanol) and carrier gas (He) for the mobile phase was applied to the gas chromatography (GC) system. A temperature program (50 °C for 2 min; increased to 250 °C at a rate of 8 °C·min^−1^; then increased to 310 °C at a rate of 30 °C·min^−1^; with 10 min of maintaining the temperature) was used. A carrier gas flow rate was set at 1 mL·min^−1^. Subsequently, 3 µL of each cyanobacteria extract was introduced into the column at an injector temperature of 250 °C. The initial oven temperature was set to 60 °C, with an automated temperature ramp of 10 °C per minute until reaching a final temperature of 280 °C. The column was held at each temperature increment for 3 min. Mass spectrometry (MS) was carried out in the electron ionisation mode with a voltage of 70 eV and an electron multiplier voltage of 1859 V. The compounds present in the samples were identified through a comparison of the mass spectrum and the retention time of each analyte with those of reference standards listed in the 2011 National Institute of Standards Journal of Food Biochemistry and Technology (NIST) library. The area percentage of each component was then determined by comparing its average peak area with the total area obtained.

### 2.9. Total Phenol Content Determination

The total phenol content of each extract was determined using the Folin–Ciocalteu assay, with gallic acid used as a standard [25]. In the procedure, 0.5 mL of the crude extract was mixed with 1.5 mL of diluted (1:10 *v*/*v*) Folin–Ciocalteu reagent. After 5 min, 1.5 mL of 7% sodium carbonate solution was added to the reaction mixture. The final volume was composed up to 10 mL with distilled water and allowed to stand for 90 min at room temperature. Absorbance was measured at 750 nm with the BioTek Synergy HT microplate reader. The total phenolic content of each extract was expressed as a gallic acid equivalent.

### 2.10. Total Flavonoid Content Determination

The total flavonoid content of each exact was determined using the aluminium chloride method described by Ordonez, et al. [26]. One millilitre of the extracts (2 mg/mL) and 4 mL of water were added into a volumetric flask (10 mL volume) and equal volume (0.3 mL) of 5% sodium nitrite and 10% aluminium chloride were added after 5 min. After 6 min of incubation at room temperature, 1 mL of 1M sodium hydroxide was added to the reaction mixture, and the final volume was totalled 10 mL with distilled water. Absorbance of the sample was measured at 510 nm, and values of flavonoid content were expressed as the quercetin equivalent.

### 2.11. In Silico Studies

Molecular docking was applied to evaluate the possibility of interactions between the structure of some beta-lactamases and the observed abundant compounds following GC-MS analysis. The selected ligands’ 3D structures were downloaded from the PubChem database. The ligand–macromolecule complex was downloaded from the Protein Data Bank in the PDB format (http://doi.org/10.2210/pdb1NYY/pdb, https://doi.org/10.2210/pdb3BM6/pdb, https://doi.org/10.2210/pdb6MGX/pdb accessed on 15 March 2024). The enzyme structure was optimised for docking using CHIMERA version 1.17.1 (UCSF, San Francisco, CA, USA); water molecules and the bound ligand was deleted, and the PDBQT format was obtained. The file was transferred to PyRx software (version 0.8, https://sourceforge.net/projects/pyrx/ accessed on 15 March 2024), where docking was carried out using AutoDock Vina 1.2.0, processed through the Vina forcefield. The ligands were docked at the position reported by the inhibitor in the PDB file at the position of its native inhibitor. The best docking conformation was visualised using Discovery Studio 24.1.0 (BIOVIA, San Diego, CA, USA), and the docking score was recorded; only the best performing ligands are depicted in the results section.

### 2.12. In Vitro Antioxidants Assay

Unless otherwise stated, butylated hydroxyanisole (BHA) and ascorbic acid (AA) were used as standards. The percentage free radical scavenging activity of the extracts was calculated from the formula: Scavenging activity (%) = [(A_control_ − A_test_)]/[(A_control_)] × 100, where A_control_ is the absorbance of the sample in the absence of inhibitor and A_test_ is the absorbance of the sample in the presence of an inhibitor.

### 2.13. 1,1-Diphenyl-2-Picryl Hydrazil (DPPH) Scavenging Activity

The DPPH radical scavenging activity of the crude extracts was investigated as described by Osunsanmi, et al. [27]. DPPH solution (0.02 mg/mL ethanol) was mixed (1:1) with the crude extracts at different concentrations (0.0–0.05). The mixture was allowed to stand for 60 min at room temperature, the absorbance was read at 517 nm, and the scavenging activity percentage was calculated.

### 2.14. 2,2-Azinobis (3-Ethylbenzothiazoline-6-Sulfonate) (ABTS+) Scavenging Activity

The ABTS scavenging activity of the extracts was evaluated as described by Sridhar and Charles [28]. Briefly, a mixture of 7 mM ABTS and 2.45 mM potassium persulfate was incubated in the dark for 16 h to generate an ABTS radical. The generated ABTS radical stock solution was diluted 60 times with ethanol to supply a working solution. Different concentrations (0.0–0.05 mg/mL) of the crude extracts were separately mixed (1:1) with ABTS* radical and incubated for 6 min at room temperature. Absorbance was read at 734 nm, and the percentage scavenging activity was calculated with the formula described above.

### 2.15. Hydroxyl Radical (^•^OH) Scavenging Activity

The hydroxyl radical scavenging activity of the cyanobacteria extracts was measured by the inhibition of deoxyribose degradation [29]. The degradation of deoxyribose by the hydroxyl radical generated was measured calorimetrically in the presence and absence of the extracts. To prepare the reaction mixture, deoxyribose (3 mM), ferric chloride (0.1 mM), EDTA (0.1 mM), ascorbic acid (0.1 mM), and H_2_O_2_ (2 mM) in phosphate buffer (pH 7.4, 20 mM) were added to various concentrations (0.0–0.05 mg/mL) of the extracts to provide a final volume of 3 mL. After incubation for 30 min at ambient temperature, trichloroacetic acid (0.5 mL, 5%) and thiobarbituric acid (0.5 mL, 1%) were added. The reaction mixture was kept in a boiling water bath for 30 min and cooled, and the absorbance was measured at 532 nm.

### 2.16. Metal Chelating Activity (Fe^2+^)

The iron chelating activity of the extracts was measured using the method of Decker and Welch [30]. In a test tube, a mixture of 0.125 mL of different concentrations (0.0–0.05 mg/mL) of the cyanobacterial extracts, 0.4 mL of distilled water, and 0.0125 mL of 2 mM iron chloride (FeCl_2_) was prepared. After 30 s, following the addition of the last reagent, the reaction was initiated by the addition of 5 mM ferrozine (0.1 mL). The mixture was well mixed and left to incubate at room temperature for 10 min. The absorbance of the mixture was spectrophotometrically read at 562 nm. Ethylenediaminetetraacetic acid (EDTA) and citric acid were used as standards.

### 2.17. Data Analysis

The data are presented as the mean ± standard deviation (SD), n = 3. Statistical differences between the groups were performed by a one-way analysis of variances (ANOVA) followed by a Dennett post hoc test ANOVA. The results were considered a statistically significant difference at *p* < 0.05.

## 3. Results

### 3.1. Isolation and Characterisation of Cyanobacteria

The isolated strains mostly exhibited filamentous morphology in liquid BG-11 media, with observable swarming when grown on solid media. The isolated strains were subsequently characterised through 16S rRNA. An NCBI BLAST analysis of the samples resulted in a 86.32% 16S rRNA gene similarity sequence match, with zero nucleotide gaps, and the predicted organisms are listed in Table 1: *Cylindrospermum alatosporum* NR125682 and *Loriellopsis cavenicola* NR117881.

### 3.2. Percentage Yield of Biomass Extraction

Table 2 shows that ethanol extracted the largest portion of the total organic chemicals of the two organisms.

### 3.3. Chemical Analysis

**Figure 1:**
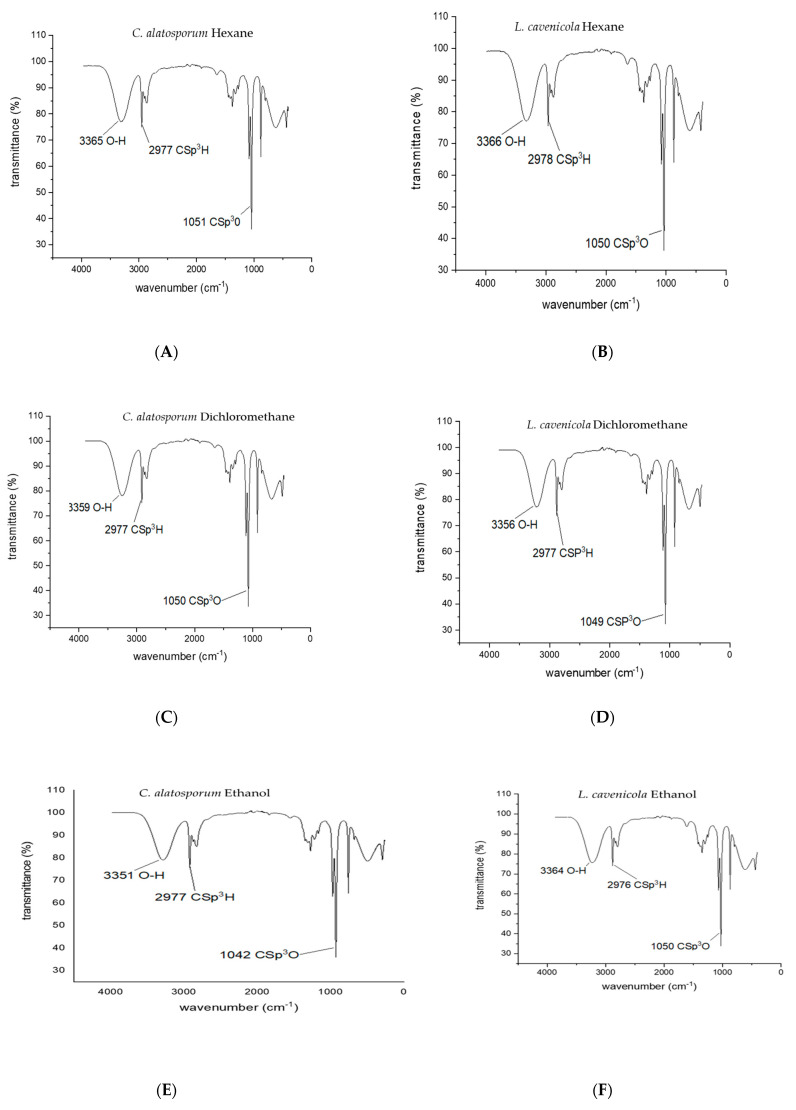
FTIR of the observed functional groups found in the extracts of *Cylindrospermum alatosporum* and *Loriellopsis cavenicola*. (**A**) Hexane *C. alatosporum* (**B**) Hexane *L. carvenicola* (**C**) Dichloromethane *C. alatosporum* (**D**) Dichloromethane *L. carvenicola* (**E**) Ethanol *C. alatosporum* (**F**) Ethanol *L. carvenicola*.

The results of the chemical analyses of the samples are presented in Figure 1 and Table 3 and Table 4.

#### 3.3.1. FTIR

The FTIR spectra of the crude extracts showed (Figure 1) similar peaks. Observed significant peak ranges were between a 1040 and 3550 cm^3^ wavenumber; a ubiquitous trend of OH stretch was observed at a peak range of 3550–3200 cm^−1^ in all extracts. Such peaks are recognised for hydroxyl (OH) and carboxyl functional groups [31]. This predicts the presence of bioactive compounds such as polyphenols and flavonoids. An alkane stretch of 3000–2840 cm^−1^, suggesting that the presence of compounds with these functional groups was also observed. C=O peaks were also observed, which points to the presence of esters, aldehydes, and carboxylic acids (1321 cm^−1^). Other prominent peaks (stretches) included C-H, CO-O-CO, and N-H.

#### 3.3.2. GC-MS

The GC-MS analysis resulted in a total of over 200 deposited compounds in the National Institute of Standards and Technology (NIST) spectral library database; the major organic compounds of the extracts are listed in Table 3 and Table 4. The two cyanobacteria contain similar groups of compounds such as aliphatic esters, phenols, and cyclic ketones; however, the dominant species were n-alkanes. The dichloromethane extracts of both cyanobacteria exhibited the most compounds, with the ethanol extracts exhibiting the fewest. Methyl-2-eicosane, 9,12,15-octadecatrien-1-ol-, and diglycerol tetranitrate were only found in the extracts of *C. alatosporum*. Regarding percentage abundance, behenyl chloride, n-hexadecanoic acid, and 10-methylundec-2-en-4-olide reported an area above 3%, with diglycerol tetranitrate being the only compound over 7%, which may attribute an antioxidant potential to the extract; this provides significance in relation to the extract’s bioactivity for this study.

#### 3.3.3. Total Phenol and Flavonoid Content

**Figure 2:**
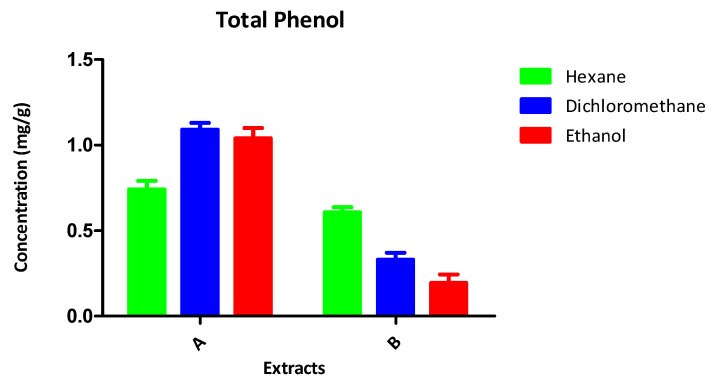
Total phenol content of the crude extracts of A: *Cylindrospermum alatosporum*; B: *Loriellopsis cavenicola*.

**Figure 3:**
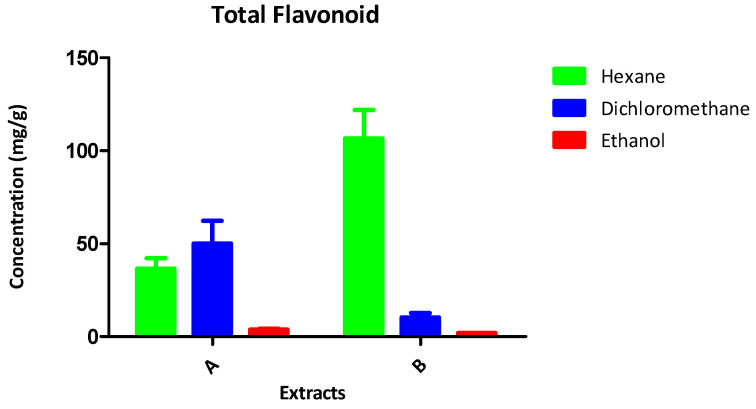
Total flavonoid content of the crude extracts of A: *Cylindrospermum alatosporum*; B: *Loriellopsis cavenicola*.

Phenols contribute to the antioxidative capacity of crude extracts. An analysis of the extracts indicates (Figure 2) that the DCM and ETOH extracts of *C. alatosporum* contained the highest amounts of phenols. The results (Figure 3) indicate that the hexane extracts of *L. cavenicola* possess an appreciable quantity of flavonoids, with the ethanol extracts having the lowest quantity. These results highlight the antioxidant profile of the extracts and potentiates their capacity as a source of novel antioxidants.

### 3.4. Antioxidants

All extracts exhibited a concentration-dependent antioxidant activity. The IC_50_ values presented in Table 5 indicate the varying degree of antioxidant efficiency. In the DPPH assay, only the ethanol displayed an IC_50_ of 6.5 ± 0.50; however, it performed poorly in the ABTS assay, where the hexane extract of *C. alatosporum* performed the best. In the metal chelating assay, the ethanol extract of *L. cavenicola* resulted in the best IC_50_ value among the extracts. It is apparent that while the extracts of *C. alatosporum* were efficient scavengers of free radicals, the extracts of *L. cavenicola* were good metal chelators.

### 3.5. In Silico Molecular Docking

**Figure 4:**
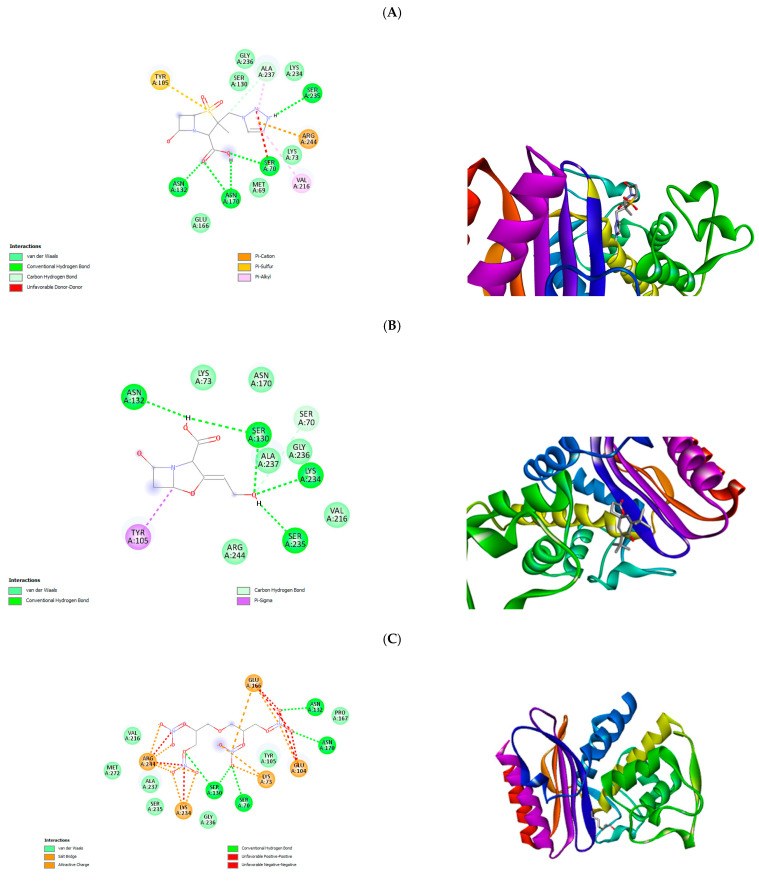

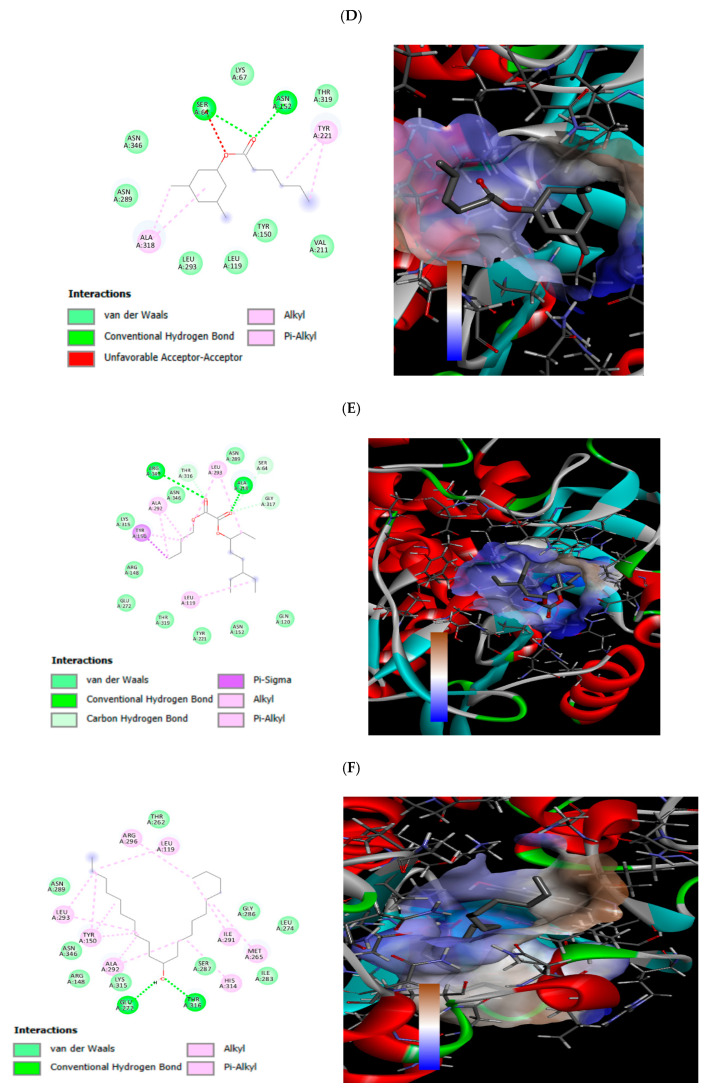

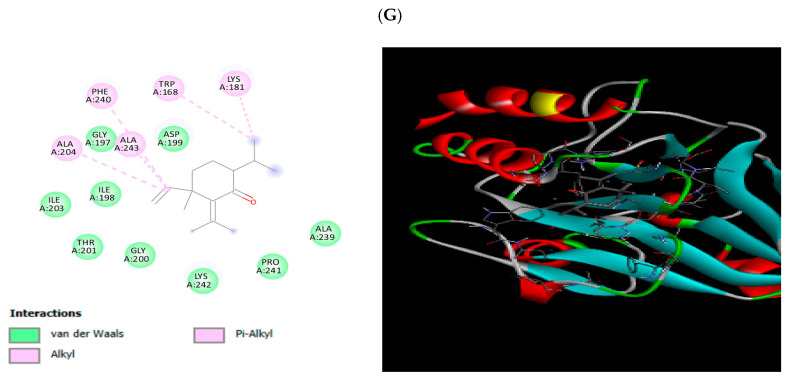
2D and 3D representation of ligand–enzyme interactions with the amino acids at the binding site of 3BM6, 1NYY, and 6MGX. (**A**–**D**) (**A**) TAZOBACTAM-1NYY complex, (**B**) CLAVULANIC ACID-1NYY complex, (**C**) DIGLYCEROL TETRANITRATE-1NYY complex, HYDRAZINECARBOXYLIC ACID, (**D**) HEXANOIC ACID, 3,5-DIMETHYLCYCLOHEXYL ESTER-3BM6 complex; (**E**) OXALIC ACID BUTYL 6-ETHYLOCT-3-YL ESTER-3BM6 complex; (**F**) HENICOSAN-11-OL-3BM6 complex; (**G**) ISOSHYOBUNONE-6MGX complex.

Table 6 contains the enzymes and their PDB ID. The most abundant compounds (area % ≥ 0.7) were selected, and the docking score of the tested compounds are presented in Table 7. It is apparent that a few of the screened compounds possess a similar good binding affinity as the standards (in blue). The images in Figure 4 revealed the 2D and 3D molecular interactions of the ligand–macromolecule complex, indicating van der Waals forces, H bonds, and electrostatic and major hydrophobic interactions. It was evident that diglycerol tetranitrate resulted in a good binding score of −6.6, which is similar to clavulanic acid (−6.7). Only the ligands with the best performing docking scores are displayed in Figure 4.

## 4. Discussion

Free radicals have been linked to a spectrum of diseases, spanning from cancer to neurological disorders [32]. Therefore, mitigating oxidative stress within biological systems serves as a pivotal mechanism in curtailing apoptosis and the autoxidation of vulnerable biological structures [33]. It has been proposed [34] that compounds demonstrating both antioxidant and antibacterial properties hold promise as potential leads for novel therapeutic drugs. Despite the existence of medications aimed at managing free radical damage and safeguarding the body against oxidative stress, the currently available drugs are notorious for their severe side effects [35]. In this study, the antioxidant activity of crude extracts from *C. alatosporum* and *L. cavenicola* (as detailed in Table 5) suggests their limited efficacy as scavengers of DPPH radicals, yet they exhibit superior scavenging abilities against ABTS radicals. Notably, the ethanol extract of *L. cavenicola* demonstrates commendable scavenging activity in the DPPH system, echoing similar trends reported by [36,37] regarding the weak activity of Nostoc extracts in the DPPH system. Conversely, the ABTS+ system presents a contrasting scenario, with the ethanol extract of *L. cavenicola* showing subpar performance. However, the remaining extracts from both cyanobacteria species, particularly those from *C. alatosporum*, display promising IC_50_ values at lower concentrations, reinforcing prior reports of potent ABTS+ scavenging by various freshwater cyanobacteria [38]. Furthermore, the ethanol extract of *C. alatosporum* and the hexane extract of *L. cavenicola* exhibit notable hydroxyl radical scavenging abilities. Several studies have linked the presence of phenols and flavonoids to the robust antioxidative capacity of natural products [39], and the high flavonoid content observed in this study can be attributed to the observed antioxidant activity. Moreover, the identified chemical constituents (detailed in Table 3 and Table 4) and the phenolic content (illustrated in Figure 2) underscore the potential of cyanobacteria as a reservoir for novel antioxidants. Interestingly, the extracts from both cyanobacteria species in this study also exhibit significant metal chelating potential.

Heavy metals disrupt protein homeostasis through enzymatic substrate competition, displacing various metallic cofactors as well as structure alteration through denaturation [40]. Cyanobacteria require a rich amount of iron for oxygenic photosynthetic processes and have evolved efficient means to outcompete other organisms for the sequestering of dissolved iron [41]. They are known to possess high iron affinity siderophores, which are synthesised for iron capture [42]; this may explain why the extracts are efficient metal chelators. Patel, et al. [43] described the strong metal chelating activity of phycocyanin (a photosynthetic pigment) from the cyanobacterium *Geitlerinema* sp., which is akin to the findings in this study. Another study by Singh, et al. [44] also reported the chelating potential of a series of cyanobacteria extracts.

Cyanobacteria have garnered attention as potential sources of antioxidants [45], and when compared, the extracts exhibit varying antioxidative capacities. *L. cavenicola* extracts demonstrate superior antioxidant activity in both the DPPH and metal chelating assays, with the ethanol crude extracts showing the strongest activity. Moreover, the robust antioxidant activity exhibited by its hexane extracts further underscores the potential of *L. cavenicola* for the development of novel antioxidants. Conversely, *C. alatosporum* demonstrates exceptional ^•^OH and ABTS+ scavenging capacity, highlighting its unique potential as a rich source of antioxidants. Notably, its hexane and ethanol crude extracts exhibit the most promising scavenging potential. Despite the dichloromethane crude extracts revealing the highest number of compounds upon GC-MS analysis, they exhibit the weakest antioxidant potency. This suggests that ethanol and hexane are preferable solvents in terms of antioxidant efficacy.

The development of in silico tools (molecular docking) has significantly quickened the screening process for metabolites, rapidly allowing for a quickened run-through and selection of promising compounds [46]. Swargiary, et al. [47] reported a study revealing the binding affinities of phytocompounds to the active sites of two crucial proteins 3-chymotrypsin- and papain-like proteases of SARSCoV2. Over 30 compounds were screened; however amentoflavone and gallocatechin gallate bound the strongest with the target proteins, making them suitable for wet lab trials. Aziz, et al. [48] synthesised a series of *N*-acyl-morpholine-4-carbothioamide derivatives and evaluated their antimicrobial and antioxidant potential; they further established the RNA-binding affinities of the compounds using docking computations to further understand the mechanistic pathway of inhibition, resulting in the identification of two potent compounds with the best docking scores. Thus, computational simulations can provide insights to molecular properties of metabolites towards the discovery of novel compounds. In this study, we evaluated the potential of the observed compounds, obtained through GC-MS analysis, to inhibit these enzymes (Table 6) using in silico techniques (molecular docking). The 2D imagery shows observable conventional hydrogen bonding with the carbonyl groups of the ligands for the screened compounds. This functional group has been attributed with the strong inhibition of beta-lactamases due to their susceptibility to hydrolysis by serine moieties at the enzyme’s active site [19]. It was noted that there are a minimum of three different types of interactions (Figure 4). The compounds present interaction, primarily, with the enzymes’ binding site through the residues LYS73, TYR105, SER130, ASN132, ASN170, VAL216, LYS234, ALA237, and ARG244. Interactions with the important residue LYS73 further potentiates the inhibition capabilities of the extract—hydrogen bond interactions—can be observed, notably formed with SER130, ASN132, and ALA237, with some hydrophobic interactions also observed; this may contribute to their better compatibility in the enzyme’s binding pocket [49]. Metallo β-lactamases are notorious for their ability to hydrolyse a wide class of b-lactam drugs, including carbapenems. In this study, the best binding affinity against the New Deli metallo-β-lactamase enzyme was obtained by isoshyobunone, recording a better affinity when compared with the standards (Table 7). Moreover, the observed recorded good binding affinity with the ser-β-lactamase enzymes suggests a potential wide range of inhibitory activity. Diglycerol tetranitrate is another compound where, despite its performance against the metallo enzyme being subpar, recorded a good affinity against the ser-β-lactamase enzymes; its superior abundance in the ethanol extract of *C. alatosporum* may indicate an inhibitory potential of the extract. The good docking score of some of the major compounds against β-lactamase potentiates the antibacterial activity of the extracts, which therefore necessitates the in vitro evaluation of the antibacterial activity of the extracts.

## 5. Conclusions

This study focuses on the preliminary analysis of the antioxidant potential of two cyanobacteria isolated from a freshwater pond in South Africa. The crude extracts exhibited significant antioxidant activity, with the estimated flavonoid content potentially contributing to this observed activity. Additionally, the docking scores obtained from certain compounds suggest a promising antibiotic potential of the crude extracts. These findings underscore the potential of cyanobacterial crude extracts as valuable sources for the discovery of novel antioxidants and antibiotic remedies. Furthermore, the isolation of pure metabolites from these crude extracts represents a promising avenue for further investigation and study.

## Figures and Tables

**Table 1:** Isolates with characterised accession numbers.

Code	Source	Organism	Acc No	% Similarity
A	Freshwater (Vulindlela)	*C. alatosporum*	NR125682	86.32%
B	Freshwater (Vulindlela)	*L. cavenicola.*	NR117881	86.32%

**Table 2:** Estimated percentage yield (dry weight basis) of biomass from both samples.

% YIELD (Dry Weight Basis)				
		Hexane	Dichloromethane	Ethanol
A	*C* *. alatosporum*	2.5 ± 0.43	1.3 ± 0.45	6.2 ± 0.26
B	*L* *. cavenicola*	1.1 ± 0.40	3.9 ± 0.66	5.0 ± 0.23

**Table 3:** Molecular formular and retention time (min) of chemical constituents identified in the extracts of *Cylindrospermum alatosporum* using gas chromatography–mass spectrometry.

Retention Time (min)	% Area	Compound Name	Molecular Formula	Molecular Weight (g/mol)	PUBCHEM ID
HEXANE					
6.469	0.10	Furan, 2,5-dihydro-2,5-dimethyl-	C_8_H_14_O_3_	158.19	86653
13.685	0.14	1,2-bis(3,5,5-trimethyl-2-cyclohexenylidene)hydrazine	C_6_H_20_N_2_Si_2_	176.41	12500123
16.598	0.17	Eicosane	C_20_H_42_	282.5	8222
19.485	0.15	2-octen-1-ol, 7-ethoxy-3,7-dimethyl-, (E)-	C_12_H_24_O_2_	200.32	5368091
21.863	0.82	9,12,15-octadecatrien-1-ol, (Z,Z,Z)-	C_18_H_32_O	264.4	5367327
27.622	0.24	Eicosane, 2-methyl-	C_21_H_44_	296.6	519146
DICHLOROMETHANE					
7.810	0.84	Decane, 2,9-dimethyl-	C_12_H_26_	170.33	517733
9.997	1.09	2,3-Dimethyldodecane	C_14_H_30_	198.39	521959
11.464	0.86	Tetradecane	C_14_H_30_	198.39	12389
12.060	1.44	Eicosane	C_20_H_42_	282.5	8222
12.114	0.94	Decane, 3,7-dimethyl-	C_12_H_26_	170.33	28468
12.403	1.76	Tetradecane, 4-methyl-	C_15_H_32_	212.4146	25117-24-2
12.548	0.84	Tridecane, 6-propyl-	C_16_H_34_	226.44	521567
12.879	1.00	Hexadecane, 2,6,11,15-tetramethyl-	C_20_H_42_	282.5	136331
14.187	0.76	Sulfurous acid, hexyl pentadecyl ester	C_21_H_44_O_3_S	376.6	6420414
14.430	1.05	Heneicosane, 11-(1-ethylpropyl)-	C_26_H_54_	366.7070	292291
14.486	0.76	Octadecane, 5-methyl-	C_19_H_40_	268.5	520183
14.821	1.11	2-methyltetracosane	C_25_H_52_	352.7	527459
15.268	0.73	Cyclohexasiloxane, dodecamethyl-	C_12_H_36_O_6_Si_6_	444.92	10911
15.666	0.88	Octadecane	C_18_H_38_	254.5	11635
16.460	0.94	Heptadecane, 2,6,10,15-tetramethyl-	C_21_H_44_	296.6	41209
16.795	1.37	N-cyclooct-4-enylacetamide	C_10_H_17_NO	167	170952-69-9
16.850	1.93	Hexadecane, 1,1-bis(dodecyloxy)-	C_40_H_82_O_2_	595.1	41920
16.900	2.27	Octadecanal, 2-bromo-	C_18_H_35_BrO	347.4	537255
16.971	2.71	2-bromotetradecane	C_14_H_29_Br	277.28	12798926
17.311	1.11	4,6-dioxatetradecane	C_12_H_26_O_2_	202	
17.360	0.75	Isoshyobunone	C_15_H_24_O	220.35	5318673
17.625	0.96	10-methyl-octadec-1-ene	C_19_H_38_	266.5	545557
17.755	1.04	Oxalic acid, butyl 6-ethyloct-3-yl ester	C_16_H_30_O_4_	286.41	6420817
17.870	2.15	(2,2,6-trimethyl-bicyclo[4.1.0]hept-1-yl)-methanol	C_11_H_20_O	168.28	535115
18.109	3.34	Behenyl chloride	C_22_H_45_Cl	345.046	545602
18.223	2.78	11-heneicosanol	C_21_H_44_O	312.6	76913
18.380	3.32	n-hexadecanoic acid	C_16_H_32_O_2_	256.42	985
19.008	0.78	Nonadecane, 2-methyl-	C_20_H_42_	282.5	137081
21.410	1.9	5,5-diethylpentadecane	C_19_H_40_	268.5	85977274
21.665	1.84	Hexadecane, 7,9-dimethyl-	C_18_H_38_	254.5	545945
21.870	0.90	Dodecane, 1,1′-oxybis-	C_24_H_50_O	354.7	20667
22.020	0.83	Hexanoic acid, 3,5-dimethylcyclohexyl ester	C_14_H_26_O_2_	226.35	565595
22.297	3.15	10-methylundec-2-en-4-olide	C_12_H_20_O_2_	196.29	21778197
23.223	1.36	Cyclohexanone, 2,2-dimethyl-5-(3-methyloxiranyl)-, [2.alpha.(R*),3.alpha.]-(.+-.)-	C_11_H_18_O_2_	182.26	534661
23.356	1.23	2-myristynoic acid	C_14_H_28_O_2_	228.37	11005
23.651	1.34	Octadecane, 3-ethyl-5-(2-ethylbutyl)-	C_26_H_54_	366.7	292285
ETHANOL					
13.790	7.15	Diglycerol tetranitrate	C_6_H_10_N_4_O_13_	346.16	30198
17.374	0.02	7,9-di-tert-butyl-1-oxaspiro(4,5)deca-6,9-diene	C_17_H_24_O_3_	276.4	545303

**Table 4:** Molecular formular and retention time (min) of chemical constituents identified in the extracts of *Loriellopsis cavenicola* using gas chromatography–mass spectrometry.

Retention Time (min)	% Area	Compound Name	Molecular Formula	Molecular Weight (g/mol)	PUBCHEM ID
HEXANE					
5.933	0.08	Hexane, 2-nitro-	C_6_H_13_NO_2_	131.17	536519
17.375	0.01	7,9-di-tert-butyl-1-oxaspiro(4,5)deca-6,9-diene	C_17_H_24_O_3_	276.4	545303
DICHLOROMETHANE					
6.510	0.80	Benzene, 1,2,4-trimethyl-	C_9_H_12_	120.19	7247
7.205	0.77	Octane, 5-ethyl-2-methyl-	C_11_H_24_	156.31	537332
7.808	0.72	Decane, 2,9-dimethyl-	C_12_H_26_	170.33	517733
9.100	1.02	Dodecane	C_12_H_26_	170.33	8182
9.849	1.30	2,4-dimethyldodecane	C_14_H_30_	198.39	521960
9.998	1.11	Decane, 3,7-dimethyl-	C_12_H_26_	170.33	28468
11.462	1.57	Tetradecane	C_14_H_30_	198.39	12389
11.574	1.03	Tetradecane, 6,9-dimethyl-	C_16_H_34_	226.44	545534
11.617	1.324	Eicosane	C_20_H_42_	282.5	8222
12.112	1.584	2,3-dimethyldodecane	C_14_H_30_	198.39	521959
12.358	1.1075	Tetradecane, 5-methyl-	C_15_H_32_	212.41	98976
12.547	1.41	Tridecane, 6-propyl-	C_16_H_34_	226.44	521567
12.776	0.83	Phenol, 2,4-bis(1,1-dimethylethyl)-	C_17_H_30_OSi	278.5	528937
13.730	0.79	Cyclooctasiloxane, hexadecamethyl-	C_16_H_48_O_8_Si_8_	593.2315	11170
14.813	1.79	Heptadecane, 2,3-dimethyl-	C_19_H_40_	268.5	537320
15.109	0.88	5,5-diethyltridecane	C_15_H_32_	212.41	41838
15.262	1.92	Cyclohexasiloxane, dodecamethyl-	C_12_H_36_O_6_Si_6_	444.92	10911
15.582	1.17	E-14-hexadecenal	C_16_H_30_O	238.41	5363106
16.420	0.71	Ethanone, 1-(2,2-dimethylcyclopentyl)-	C_9_H_16_O	140.22	537088
16.964	0.92	Decane, 2,3,5,8-tetramethyl-	C_14_H_30_	198.39	545611
17.281	2.08	Docosane, 2,4-dimethyl-	C_24_H_50_	338.7	538282
18.866	0.81	1-heptadecene	C_17_H_34_	238.5	23217
27.481	1.16	Eicosanoic acid, 2-hydroxyethyl ester	C_20_H_40_O_3_	328.5298	111-60-4
ETHANOL					
7.995	0.03	Nonanal	C_9_H_18_O	142.24	31289
17.375	0.03	7,9-di-tert-butyl-1-oxaspiro(4,5)deca-6,9-diene-2,8	C_17_H_24_O_3_	276.4	545303

**Table 5:** IC_50_ of the antioxidant activities of the crude extracts (H: hexane; D: dichloromethane; E: ethanol GA: gallic acid; AA: ascorbic acid; BHA: butylated hydroxylanisole; EDTA: ethylenediaminetetraacetic acid).

IC_50_ (µg/mL)				
*C. alatosporum*				
EXTRACT	DPPH	ABTS	^•^OH	FE^2+^
H	ND	6.6 ± 0.62	48.6 ± 1.10	69.3 ± 3.24
D	ND	6.8 ± 0.82	37.2 ± 2.32	72.3 ± 2.22
E	ND	6.9 ± 0.69	6.4 ± 0.59	45 ± 0.78
***L. cavenicola***				
H	ND	7.1 ± 0.91	6.8 ± 0.48	44.3 ± 2.25
D	ND	9.5 ± 0.32	18.2 ± 1.66	51.3 ± 2.65
E	6.5 ± 0.50	ND	15 ± 2.15	44.7 ± 5.60
AA	4.1 ± 0.48	4.7 ± 0.37	ND	ND
BHA	4.3 ± 0.55	4.2 ± 0.22	ND	ND
GA	ND	ND	ND	ND
EDTA	ND	ND	ND	75.7 ± 5.40

Values are presented as the mean ± standard deviation (n = 3). ND = not determined. No observable statistical significance between the extracts and the respective assay standard.

**Table 6:** Enzymes and their Protein Data Bank ID.

Protein/Receptor	PDB ID
TEM-1 beta-lactamase	1NYY
AmpC beta-lactamase	3BM6
New Deli Metallo-beta-lactamase	6MGX

**Table 7:** Docking score of tested compounds against beta-lactamases.

PUBCHEM ID	Compound Name	Binding Affinity (Kcal/mol)		
		3BM6	6MGX	1NYY
11635	Octadecane	−5.3	−5.0	−4.1
12389	Tetradecane	−4.6	−4.9	−4.1
12798926	2-bromotetradecane	−4.8	−4.3	−4.3
136331	2,6,11,15-tetramethylhexadecane	−5.9	−4.9	−4.5
137081	Nonadecane, 2-methyl-	−4.4	−5.2	−4.3
20667	Dodecane, 1,1′-oxybis-	−4.6	−5.0	−4.5
21778197	10-methylundec-2-en-4-olide	−5.7	−5.8	−5.5
28468	Decane, 3,7-dimethyl-	−4.5	−4.9	−4.4
292285	Octadecane, 3-ethyl-5-(2-ethylbutyl)-	−5.7	−5.8	−4.6
292291	Heneicosane, 11-(1-ethylpropyl)-	−4.9	−5.6	−5.0
30198	Diglycerol tetranitrate	−6.4	−4.9	−6.6
324386	2-myristynoic acid	−5.5	−4.7	−5.2
41209	Heptadecane, 2,6,10,15-tetramethyl-	−5.4	−5.9	−5.0
41920	Hexadecane, 1,1-bis(dodecyloxy)-	−5.0	−5.5	−4.0
520179	Tetradecane, 4-methyl-	−4.8	−4.7	−4.6
520183	Octadecane, 5-methyl-	−4.5	−4.5	−4.4
521567	Tridecane, 6-propyl-	−4.3	−5.0	−4.4
521959	2,3-dimethyl-dodecane	−5.7	−5.8	−4.8
527459	Tetracosane, 2-methyl-	−4.7	−5.9	−4.4
5318673	Isoshyobunone	−5.9	−6.3	−6.2
534661	2,2-dimethyl-5-(3-methyl-2-oxiranyl)cyclohexanone	−5.5	−5.8	−6.3
535115	(2,2,6-trimethyl-bicyclo[4.1.0]hept-1-yl)-methanol	−4.9	−5.7	−5.7
5363538	N-cyclooct-4-enylacetamide	−5.3	−5.3	−5,6
537255	Octadecanal, 2-bromo-	−5.4	−5.1	−4.3
545557	10-methyl-1-octadecene	−5.6	−5.0	−4.4
545602	Behenyl chloride	−5.2	−5.3	−4.1
545945	Hexadecane, 7,9-dimethyl-	−5.5	−4.6	−4.4
565595	Hexanoic acid, 3,5-dimethylcyclohexyl ester	−6.0	−5.7	−5.7
6420414	Sulfurous acid, hexyl pentadecyl ester	−5.0	−4.8	−4.6
6420817	Oxalic acid, butyl 6-ethyloct-3-yl ester	−6.0	−4.6	−5.6
76913	Henicosan-11-ol	−6.1	−5.1	−4.7
85977274	5,5-diethylpentadecane	−5.1	−4.9	−4.4
91691637	4,6-dioxatetradecane	−5.0	−4.2	−4.6
985	Hexadecanoic acid	−5.8	−5.2	−4.7
123630	Tazobactam	−6.8	−5.8	−7,4
23217	Heptadec-1-ene	−4.8	−4.8	−4.3
517733	Decane, 2,9-dimethyl-	−5.5	−5.7	−4.2
521960	2,4-dimethyl-dodecane	−4.6	−5.1	−5.5
5280980	Clavulanic acid	−6.0	−5.7	−6,7
5363106	14-hexadecenal, (E)-	−4.9	−4.4	−4.7
537088	Ethanone, 1-(2,2-dimethylcyclopentyl)-	−4.7	−5.7	−4.9
537320	2,3-dimethyl-heptadecane	−4.7	−5.3	−4.4
537332	Octane, 5-ethyl-2-methyl-	−4.8	−4.6	−4.3
538282	Docosane, 2,4-dimethyl-	−5.5	−5.7	−4.5
538813	Eicosanoic acid, 2-hydroxyethyl ester	−5.8	−5.6	−5.0
545534	Tetradecane, 6,9-dimethyl-	−5.2	−4.8	−4.5
545611	Decane, 2,3,5,8-tetramethyl-	−4.9	−5.3	−4.7
7247	Benzene, 1,2,4-trimethyl-	−5.2	−5.5	−4.9
7311	Phenol, 2,4-bis(1,1-dimethylethyl)-	−5.9	−5.9	−6.0
8182	n-dodecane	−4.0	−4.6	−3.9
8222	n-eicosane	−5.3	−5.4	−4.1
85977273	5,5-diethyltridecane	−4.5	−4.6	−4.4
98976	Tetradecane, 5-methyl-	−4.5	−5.0	−4.3

## Data Availability

The data used to support the findings of this study are available from the corresponding author upon request.
